# Psychosocial Processes and Mechanisms of Risk and Protection

**Published:** 2004

**Keywords:** underage drinking, adolescent, cause of AODU (alcohol and other drug use), risk factors, protective factors, AOD expectancies, predictive factor, AOD use behavior, personality theory of AODU, psychosocial environment, personality trait, negative emotionality, positive emotionality

## Abstract

Psychosocial research on adolescent drinking includes studies of personality and the impact of particular personality traits on drinking risk, expectancies (that is, the effects someone expects after drinking alcohol), and cognitive development. Although studies involving adolescents have not identified specific sets of personality traits that uniquely predict alcohol use, some traits have been shown to be associated with heavy alcohol use and alcohol use disorders. These traits include disinhibition or poor self-regulation, impulsiveness and aggression, novelty-seeking, and negative affectivity. Externalizing behaviors in childhood and early adolescence have been found to predict alcohol use disorders in early adulthood, as have certain internalizing behaviors. This article examines the theories and psychosocial processes thought to underlie underage drinking.

## Overview

The interactions among alcohol-related genes, biological development, and environment play out in the psychological processes underlying adolescent decisions to drink or to abstain from drinking. Psychosocial research on adolescent drinking encompasses studies of personality and the impact of particular personality traits on drinking risk, expectancies (the effects someone expects from drinking alcohol), and cognitive development.

As is true for adults, studies involving adolescents have repeatedly failed to find specific sets of personality traits that uniquely predict alcohol use. In addition, adolescence is a period of change, and personality is not as stable as it will be in adulthood. Nonetheless, some personality traits have been shown to be associated with heavy alcohol use and alcohol use disorders in adolescents. These traits include disinhibition or poor self-regulation, impulsiveness and aggression, and novelty-seeking. Longitudinal studies have found that externalizing behaviors in childhood and early adolescence predict alcohol use disorders in early adulthood.

Negative emotionality—depression and anxiety—also have been found to predict alcohol problems. Adolescents in this case may use drinking as a coping strategy.

Expectancies about the effects of alcohol are measurable in children before they ever begin to drink. Alcohol-related expectancies influence how early a child will begin to drink and how much she or he will drink at that point. Research suggests that people who have expectancies of more positive experiences from drinking tend to drink more than others and are at highest risk for excessive drinking. Research is looking into the neural processes underlying expectancies and exactly how they drive behavior.

An almost universal theme whenever adolescent drinking is addressed relates to how adolescents think and make decisions about the world around them. Despite much literature suggesting that adolescents have not yet reached full maturity in their cognitive processing, when called upon to make reasoned decisions using abstract processes, they generally do as well as adults. Differences in decisionmaking appear between adults and adolescents in situations that may have social or emotional overtones. Like adults, adolescents may vary their judgments based on social context, but the contexts that encourage such decisionmaking differ for adults and adolescents.

With this in mind, adolescent thinking and decisionmaking may be best understood as fully developed for the purpose for which they evolved: to deal with the tremendous transitions that humans face at this stage of life. The goal for research is how to integrate this emerging understanding of adolescence with the need to reduce adverse outcomes.

Many factors play a part in the development of adolescent drinking. Comprehensive theories on (or models of) the development of adolescent drinking create a framework for understanding and testing ideas about how multiple factors interact to lead to problems with alcohol. One of the goals of NIAAA’s underage drinking initiative is to stimulate the synthesis and testing of new and comprehensive models for adolescent drinking within a developmental framework.

## Personality and Alcohol Problems in Youth

Although the relationship between personality and alcohol use disorders (AUDs) has been extensively studied in older adolescents and adults (Sher et al. 1999), far less research has been conducted with respect to personality and alcohol involvement earlier in adolescence. Indeed, personality in adolescence in general is much less developed as a research area than it is in adults. This is probably due in part to the tendency among researchers interested in individual differences in infancy and childhood to focus on temperamental traits that are thought to represent very basic tendencies in a person’s response to the environment. These temperamental traits are highly heritable and are usually assessed by parents or other adults. In contrast, researchers focusing on adults tend to look at more complex traits obtained through self-reports. Thus, the period of adolescence (especially early adolescence) sits at the junction of research traditions on childhood temperament and adult personality, which may be why this age has not received a greater amount of attention and development.

**Cognitive Development and Adolescent Decisionmaking**

An almost universal theme whenever adolescent drinking is addressed relates to how adolescents think and make decisions about the world around them. As perhaps a reflection of this emphasis, the recent National Research Council and Institute of Medicine report on underage drinking includes two separate background papers on this topic (Halpern-Felsher and Biehl 2004; Jacobs 2004), which provide a foundation for this overview. The classic conclusion in this domain is that adolescents have not yet achieved full maturity of their cognitive processing and that they are more likely than adults to make risky decisions. A large body of literature exists containing many variations on the theoretical underpinnings for this conclusion. We will not attempt to review this material in any depth for this article, but a few ideas that have been central to this conclusion are worth noting. One is that the transition to the Piagetian concept of “formal operations” in thinking style has not yet taken place. Another view is that adolescents are very egocentric and feel invulnerable to harm because of their perceived uniqueness (Elkind 1967, 1978). Still another view is that adolescents use rational (reason-based) thinking in fewer situations than adults and depend more on intuitive processing that involves cognitive heuristics and judgment biases (Agnoli 1991; Barrouillet et al. 2002; Davidson 1995; Jacobs and Potenza 1991).

Social considerations are a potentially important factor; that is, adolescents are understood to be very interested in their social standing among their peers and therefore are more vulnerable to decisionmaking that relies heavily on what other adolescents are doing. Related to this notion is that personal identity is less well established in adolescence, with the result that young people are more influenced by what they perceive others around them to be doing. A more recent version of this theme is based on neurological development: the neural substrate for emotional behavior develops in advance of the more frontal, rational decisionmaking portion of the brain (Luna and Sweeney 2004). Other versions of this theme are more specific to the alcohol field. For example, a recurrent view is that young people systematically overestimate the frequency and quantity of drinking being undertaken by their peers (DeJong 2002; Aas and Klepp 1992; Beck and Treiman 1996).

Although there may be aspects of the above views that warrant further consideration, our understanding of the general domain of cognitive development and risk taking also is evolving in a way that offers an entirely different perspective on these issues. First of all, adolescent decisionmaking generally has not been found to be inferior to that of adults. When called upon to make reasoned decisions using abstract processes, adolescents generally do as well as adults. In many instances, however, both adolescents and adults perform poorly; unless reasoned thinking is somehow explicitly required in a particular circumstance, both adolescents and adults will use the more intuitive style of problem solving, leading to a generally equal rate of errors. It is evident, therefore, that we cannot entirely attribute the apparent riskiness of adolescence to fundamental differences in problem solving between adolescents and adults. Where differences do appear, however, is in real-world situations that may have social and/or emotional overtones. In these situations, adolescents often do not make the same choices as adults. When adolescent decisionmaking is analyzed closely for underlying processes, the results suggest that adolescents do not use appropriate base rate information about peers but instead use social heuristics or “rules of thumb” rather than actual counts of behavioral frequencies to vary their judgments based on particular contexts (e.g., to use different judgment rules when they are with social peers with high standing in the peer group, etc.) (Gardner and Steinberg 2005; Steinberg 2004).

It is important to appreciate that adults also make decisions and judgments based on these biases. It is just that the particular content areas that accentuate biased decisionmaking may be different (e.g., appearance vs. role performance), as may the contexts in which these biases most often occur. And in some domains, decision-making actually may become more biased (i.e., based on social heuristics) as children mature into adolescence. It is clearly not the case, therefore, that humans become more “rational” with age in a linear fashion.

Hence, although it is clearly appropriate to apply cognitive development and decisionmaking models to the understanding of adolescent drinking, these models should be informed by the emerging picture of adolescent development. For example, it might be best to broaden our conceptualization of adolescent thinking and not assume that adolescent thinking is deficient in some sense relative to adult decisionmaking, only to arrive at “full” levels of cognition with adulthood. Instead, adolescent thinking and decisionmaking may be better understood as fully developed for the purpose for which they evolved; that is, to deal with the tremendous transitions that humans face at this stage of life. How to integrate this emerging understanding of adolescence with the need to reduce adverse outcomes from drinking is a critical question.

ReferencesAasHKleppKIAdolescents’ alcohol use related to perceived normsScandinavian Journal of Psychology333153251992128782410.1111/j.1467-9450.1992.tb00920.xAgnoliFDevelopment of judgmental heuristics and logical reasoning: Training counteracts the representativeness heuristicCognitive Development61952171991BarrouilletPMarkovitsHQuinnSDevelopmental and content effects in reasoning with causal conditionalsJournal of Experimental Child Psychology8123524820021188408910.1006/jecp.2001.2652BeckKHTreimanKAThe relationship of social context of drinking, perceived social norms, and parental influence to various drinking patterns of adolescentsAddictive Behaviors216336441996887676210.1016/0306-4603(95)00087-9DavidsonDThe representativeness heuristic and conjunction fallacy effect in children’s decision-makingMerrill-Palmer Quarterly413283461995DeJongWThe role of mass media campaigns in reducing high-risk drinking among college studentsJournal of Studies on AlcoholSuppl 1418219220021202272410.15288/jsas.2002.s14.182ElkindDEgocentrism in adolescenceChild Development381025103419675583052ElkindDUnderstanding the young adolescentAdolescence131271341978GardnerMSteinbergLPeer influence on risk taking, risk preference, and risky decision making in adolescence and adulthood: An experimental studyDevelopmental Psychology4162563520051606080910.1037/0012-1649.41.4.625Halpern-FelsherBLBiehlMDevelopmental and environmental influences on underage drinking: A general overviewNational Research Council and Institute of MedicineReducing Underage Drinking: A Collective ResponsibilityBonnieRJO’ConnellMEWashington, DCNational Academies Press2004402416Available online at: http://www.nap.edu/books/0309089352/html20669473JacobsJEPerceptions of risk and social judgments: Biases and motivational factorsNational Research Council and Institute of MedicineReducing Underage Drinking: A Collective ResponsibilityBonnieRJO’ConnellMEWashington, DCNational Academies Press2004417436Available online at: http://www.nap.edu/books/0309089352/html20669473JacobsJEPotenzaMTThe use of judgment heuristics to make social and object decisions: A developmental perspectiveChild Development621661781991LunaBSweeneyJAThe emergence of collaborative brain function: fMRI studies of the development of response inhibitionAnnals of the New York Academy of Sciences1021269309200410.1196/annals.1308.03515251900SteinbergLRisk taking in adolescence: What changes, and why?Annals of the New York Academy of Sciences1021515820041525187310.1196/annals.1308.005

Numerous personality traits have been described in the literature, but research suggests that most of these can be subsumed by a handful of higher order traits. Researchers disagree on the number of these higher order traits, but current influential models are usually defined as either “Big Three” (Eysenck 1990; Tellegen 1985) or “Big Five” (e.g., Costa and McCrae 1992; Digman 1989, 1990; Goldberg 1990, 1992). Big Three approaches typically describe their factors as representing: (1) negative emotionality or neuroticism, (2) positive emotionality or sociability or extraversion, and (3) impulsivity or behavioral undercontrol or (lack of ) constraint. Both negative emotionality/neuroticism and positive emotionality/sociability/extraversion have their counterparts in Big Five approaches, but impulsivity or behavioral under-control or (lack of ) constraint appears primarily to be reflected in the Big Five trait of (or lack of ) conscientiousness as well as in the Big Five traits of (low) agreeableness and neuroticism. In addition, a higher order trait referring to either “openness to experience” or “intellect” (depending upon the personality system) also emerges. Research in older adolescents and adults strongly suggests that traits related to behavioral undercontrol are the most strongly associated with both alcohol use and alcohol use disorders, whereas traits associated with negative emotionality are somewhat less important (Sher et al. 1999). Existing research suggests that these basic findings generalize to younger adolescents, but the evidence base at present is somewhat underdeveloped (Caspi et al. 2005).

### Stability of Adolescent Personality

Because adolescence is a period of dramatic physical, social, and interpersonal change, it is reasonable to believe that the structure and stability of adolescent personality differs in important ways from the structure and stability of adult personality. Much of the current research on adolescent personality focuses on the rank order stability of various personality traits from adolescence through adulthood. Surprisingly, amidst a preponderance of significant life changes, the rank order stability of adolescent personality remains remarkably high. Specifically, correlations across time among Big Five personality traits range from .47 to .51 throughout adolescence (Roberts and DelVecchio 2000; Shiner 2005). Importantly, however, these relatively high correlations are far from unity. Moreover, personality stability appears to increase throughout adulthood (with peak correlations after age 50), suggesting that personality during adolescence is relatively kinetic and unsettled. Other studies of adolescent personality focus on differing levels of various traits in adult and adolescent populations (Caspi et al. 2005). These studies typically find that traits related to agreeableness, conscientiousness, dominance, and openness to experience increase from adolescence to middle adulthood, whereas traits related to neuroticism and sociability decrease during the same period (Caspi et al. 2005).

It also should be noted that the structure and correlates of alcohol disorders tend to change across the life span. For example, a growing body of research suggests that different AUD symptoms have different meanings at different developmental levels (Chung et al. 2005; O’Neil and Sher 2000). Moreover, there are significant age-related changes in the prevalence and course of AUDs (Grant et al. 1994; Hasin and Grant 2004). It will be important for future research to examine adolescent alcohol and other drug use from a developmental perspective with a focus on how changes in the structures of both personality and AUDs interact across the life span.

The relationship between personality and adolescent alcohol use is complex. As is true for adults, studies involving adolescents have repeatedly failed to find specific constellations of personality traits that uniquely predict alcohol use (e.g., an “alcoholic personality”). In addition, a dearth of research has examined the stability and structure of adolescent personality traits. At the same time, however, certain personality traits have been consistently associated with adolescent drinking patterns.

#### Disinhibition

Traits related to disinhibition or poor self-regulation have been shown to predict both heavy alcohol use and alcohol use disorders in adolescent samples. For example, Soloff and colleagues (2000) found higher levels of impulsiveness and aggression among a sample of young adults with AUDs than among age-matched control subjects. Similarly, Gabel and colleagues (1999) found that novelty-seeking predicted alcohol and other drug dependence symptoms both in a sample of treatment-referred male adolescents and in age-matched control subjects. Other studies have found relationships between alcohol problems and behavioral undercontrol (King and Chassin 2004), rebelliousness (Brook et al. 1995), low constraint (Chassin et al. 2004), low harm avoidance (Jones and Heaven 1998), and a host of other disinhibited traits (Colder and O’Connor 2002; Moss and Kirisci 1995; Colder and Chassin 1997). Moreover, impulse control disorders such as conduct disorder, oppositional defiant disorder, and borderline personality disorder are highly comorbid with alcohol and other drug pathology in adolescents, suggesting the possibility of shared etiological pathways (Clark et al. 1997; Gabel et al. 1999; Serman et al. 2002; Chassin et al. 2002). Indeed, research by Slutske and colleagues (2002) indicates that much of the high genetic correlation between conduct disorder and adult alcohol use disorders is associated with personality traits related to behavioral disinhibition.

Additionally, adolescents at high risk for the development of alcohol use disorders because of a family history of alcoholism have been shown to be characterized by high levels of disinhibition. Specifically, several researchers have found high levels of impulsive and externalizing behaviors among adolescent children of alcoholics (COAs). For example, Clark and colleagues (1999) found elevated rates of antisocial disorders among early adolescent COAs compared with their non-COA peers. Similarly, Sher and colleagues (1991) found high levels of behavioral undercontrol among late adolescent college students with family histories of alcoholism. It is important to note, however, that a number of studies have failed to find associations between COA status and externalizing behavior (Alterman et al. 1986; Pihl et al. 1990). Moreover, COA–disinhibition relationships tend to be small and may be the result of shared third variables such as parental antisociality or chaotic family environments (Sher 1997). Despite these methodological concerns, however, the majority of high-risk studies support COA/non-COA behavioral differences across a variety of methodologies and samples (Pihl et al. 1990).

Perhaps the most informative data on disinhibition and adolescent alcohol use come from longitudinal studies. These studies often find that externalizing behaviors assessed in childhood/early adolescence predict AUDs in early adulthood. For example, Cloninger and colleagues (1988) used teacher ratings of 431 schoolchildren assessed at age 11 to predict alcohol abuse at age 27. Results showed that high novelty-seeking and low harm avoidance measured during childhood predicted alcohol abuse during adulthood. Similarly, Caspi and colleagues (1997) found that aggression, alienation, low harm avoidance, low control, and low social closeness assessed at age 18 predicted alcohol dependence at age 21. Using a somewhat different methodology, Iacono and colleagues (2002) found that 17-year-olds with attenuated P3 event-related potential amplitudes (often considered a marker of externalizing disorders) were at risk for alcohol and other drug use disorders at age 20. Moreover, studies using growth mixture models suggest that disinhibited traits measured during adolescence are associated with problematic alcohol and other drug use trajectories (Chassin et al. 2002; Chassin et al. 2004).

**Table:** A Snapshot of Research Findings on Adolescent Alcohol Use Disorders and Personality Traits

Findings	Study
	
***Disinhibition/Poor Self-Regulation***	
Higher rates of impulsiveness and aggression were found in young adults with alcohol and other drug use disorders than among an age-matched control group.	Soloff et al. 2000

Higher rates of novelty-seeking predicted substance dependence symptoms in both a sample of treatment-referred male adolescents and age-matched control subjects.	Gabel et al. 1999

Alcohol problems were found to be associated with:	
• Behavioral undercontrol	• King and Chassin 2004
• Rebelliousness	• Brook et al. 1995
• Low constraint	• Chassin et al. 2004
• Low harm avoidance	• Jones and Heaven 1998
• Other disinhibited traits	• Colder and O’Connor 2002; Moss and Kirisci 1995; Colder and Chassin 1997

Impulse control disorders such as conduct disorder, oppositional defiant disorder, and borderline personality disorder are highly comorbid with substance use disorders in adolescents.	Clark et al. 1997; Gabel et al. 1999; Serman et al. 2003; Chassin et al. 2002

Adolescents at high risk for development of alcohol use disorders because of family history have been found to have high rates of disinhibition:	
• Early adolescent children of alcoholics had higher rates of antisocial disorders than non-COA peers.	• Clark et al. 1999
• College students showed higher levels of behavioral undercontrol.	• Sher et al. 1991
BUT:	
• Some studies have failed to find associations between COA status and externalizing behavior.	• Alterman et al. 1986; Pihl et al. 1990
• Relationships between COA status and disinhibition are small and may be caused by third variables such as parental antisociality or chaotic home environments.	• Sher 1997

High novelty-seeking and low harm avoidance among 11-year-olds predicted alcohol abuse during adulthood.	Cloninger et al. 1988

Aggression, alienation, low harm avoidance, low control, and low social closeness at age 18 predicted alcohol dependence at age 21.	Caspi et al. 1997

Disinhibited traits measured during adolescence are associated with problematic alcohol and drug use trajectories.	Chassin et al. 2002, 2004

***Negative Affectivity/Neuroticism***	
• Negative affect was associated with both heavy drinking and frequency of alcohol use in a sample of early adolescents.	• Colder and Chassin 1993
• A later assessment of the same sample found that negative affect predicted alcohol use and interacted with impulsivity to predict both alcohol use and alcohol-related impairment.	• Colder and Chassin 1997
• High rates of negative emotionality were found among nonalcoholic adolescents with a parental history of alcoholism.	• Elkins et al. 2004
• Baseline anxiety disorders predicted the subsequent onset and course of AUDs in an adolescent sample.	• Zimmerman et al. 2003
BUT:	
• Some researchers have found that relationships between negative affectivity and alcohol use problems were not significant.	• Brook et al. 1986; White et al. 1986
• Some research with adults indicates that negative emotionality is a result rather than a cause of alcohol pathology.	• Sutherland 1997
• Recent studies suggest that negative emotionality is only predictive of alcohol problems among adolescents who use drinking as a coping strategy.	• Cooper 1994; Newcomb et al. 1988

SOURCE: www.MonitoringtheFuture.org.

#### Neuroticism/Negative Affectivity

Negative affectivity also has been shown to predict alcohol problems in adolescent samples. For example, Colder and Chassin (1993) found that negative affect was associated with both heavy drinking and frequency of alcohol use in a sample of 452 early adolescents. In a later assessment of the same sample, Colder and Chassin (1997) found that negative affect (1) predicted alcohol use and (2) interacted with impulsivity to predict both alcohol use and alcohol-related impairment. Other cross-sectional studies have yielded similar findings (Labouvie et al. 1990; Loukas et al. 2000; Krueger 1999).

High-risk and prospective studies also suggest a relationship between negative affectivity and AUDs. For example, Elkins and colleagues (2004) found high rates of negative emotionality among nonalcoholic adolescents with a parental history of alcoholism. Similarly, Zimmerman and colleagues (2003) found that baseline anxiety disorders predicted the subsequent onset and course of AUDs in an adolescent community sample. It should be noted, however, that several researchers have found nonsignificant relationships between negative affectivity and alcohol use problems (Brook et al. 1986; White et al. 1986). In addition, some research using adult samples indicates that negative emotionality is a consequence, rather than a cause, of alcohol pathology (Sutherland 1997). Interestingly, other studies suggest that negative emotionality is only predictive of alcohol problems among adolescents who use drinking as a coping strategy (Cooper 1994; Newcomb et al. 1988). More research is needed to clarify the mediational role of drinking motives in the negative affectivity/alcohol problem relationship.

### Functional Relations Between Personality and Drinking

Within the adult literature, there has clearly been a move away from simply demonstrating correlations between specific personality traits and alcohol involvement and toward specifying the ways that personality could affect alcohol involvement through plausible mediating mechanisms. To date, several mediating models have been proposed, including those which suggest that certain personality traits are related to specific drinking motivations (e.g., anticipated social facilitation or self-medicating motives), deviant socialization into heavy-drinking peer groups, and pharmacological vulnerability to reinforcement from alcohol (Sher et al. 1999). In turn, these mediators are believed to account, in part, for the association between personality and alcohol involvement. Available data suggest that these same general mechanisms are applicable to adolescent drinking, although there is probably some developmental specificity, with deviant socialization being a more important mechanism during adolescence.

## Alcohol Expectancies and Related Concepts

Although the expectancy concept^1^ has been used across a wide variety of domains within behavioral science, it has received particular attention within the alcohol field (see Goldman et al. 1987, 1999). And within the alcohol field, one point of special focus has been expectancy and the development of drinking patterns from childhood through adolescence and into young adulthood (Christiansen et al. 1989; Dunn and Goldman 1996, 2000; Gaines et al. 1988; Lang and Stritzke 1993; Miller et al. 1990; Smith et al. 1995; Zucker et al. 1995). The original application of the term “expectancy” within the alcohol field was to experimental conditions in balanced-placebo studies of the psychopharmacological effects of alcohol. In these conditions, the participants were led to believe that they were drinking alcohol but actually may have received either alcohol or a placebo beverage, or were led to believe that they were drinking a nonalcoholic beverage but actually received a beverage which may have contained alcohol (see Goldman et al. 1987). In general, according to these studies, the behavior displayed after alcohol consumption often was as much a function of the expectation (instructional set) that alcohol had been administered as it was related to what actually was administered (up to particular dosage levels).

In the early 1980s, reports appeared in the literature describing studies in which participants responded to questionnaires that inquired directly about their expectancies—that is, the effects they anticipated if they were to consume alcohol (e.g., alcohol makes it easier to talk to people, to pick a fight, to be sexually responsive; see review by Goldman et al. 1999). Since that time, hundreds of studies of this type have been completed, with the consistent finding that particular response patterns on these questionnaires correlate substantially with self-reported alcohol use, and that these same response patterns can be used to predict later drinking over a time-lagged (longitudinal) period. Among those expectancies these studies most often identified as being related to higher levels of drinking are those for enhanced social/sexual functioning and positive emotional outcomes. Summed across studies, the data suggest that participants who hold more expectancies for positive/arousing outcomes from drinking, or more strongly endorse such expectancies on a Likert scale (with which respondents rate the extent to which they agree or disagree with a given statement), tend to drink more.

Of special relevance to the present review is that these expectancies are measurable in children before they ever begin to drink (Christiansen et al. 1989; Dunn and Goldman 1996, 1998; Gaines et al. 1988; Lang and Stritzke 1993; Miller et al. 1990; Noll et al. 1990; Smith et al. 1995; Stacy et al. 1991; Zucker et al. 1995). And expectancies, even at this early point in development, are related to how early children will begin to drink and to how much they will drink when they begin drinking. It is important to note that some assessment devices for obtaining expectancies from children have been accommodated to their developmental level (in terms of reading level or mode of presentation; see Dunn and Goldman 1996, 1998; Miller et al. 1990). It is also important to note that the rudimentary development of expectancies has been established even before the start of elementary school (Noll et al. 1990). Perhaps most interesting is the finding that children in general shift from a primary emphasis on the negative or adverse effects of drinking alcohol before about age 9 to a primary emphasis on the positive and arousing effects of alcohol by about age 13 (Dunn and Goldman 1996, 1998). Those at highest risk for excessive drinking show the largest emphasis on positive/arousing effects. Although speculation as to the processes that might cause this developmental shift has been offered, the actual causes remain to be determined.

It is, of course, quite possible to simply regard alcohol expectancies as a list or inventory of expected effects of alcohol. The history of the use of the expectancy concept in several different venues in behavioral science suggests, however, that expectancies can be an active process variable, with causal or mediational influence over behavioral output. For example, expectancy explanations have been applied to a wide variety of clinical phenomena including pain reduction, placebo effects, psychotherapy, and hypnosis (see Kirsch 1999), and to explanations of other psychological phenomena including comparative judgment (Ritov 2000), music appreciation (Krumhansl and Toivaine 2000), and even operant (Dragoi and Staddon 1999) and classical conditioning (Rescorla and Wagner 1972).

Research also has linked underlying neural phenomena with expectancy. For example, neuronal signaling involving the neurotransmitter dopamine in the nucleus accumbens (part of the brain’s reward/reinforcing circuitry) has been noted to encode expectations about external rewards (Kupfermann et al. 2000). Emotional responses, centering on the operation of the amygdala (another component of the brain’s reward circuitry), also can be linked to expectancy (Kupfermann et al. 2000). Within the alcohol and substance abuse fields, expectancy has been understood to reflect differential dopamine activity in genetically selected animal lines (Katner et al. 1996) and the manner in which well-established individual differences in the metabolism of alcohol influence alcohol consumption (McCarthy et al. 2000).

Viewed in this way, alcohol expectancies can have significant implications for how people make choices to use alcohol and about how much to use. Six increasingly stringent levels of evidence support a causal role (see Goldman 2002): (1) expectancies correlate with reports of alcohol use, accounting for up to 50 percent of the variance in drinking outcomes (Earlywine 1994; Leigh and Stacy 1993); (2) expectancies are found in children before drinking begins and predict drinking prospectively into adolescence and young adulthood (Dunn and Goldman 1996, 1998; Zucker et al. 1995); (3) expectancies increase with drinking experience (Smith et al. 1995); (4) at the point in life at which drinking decreases in many people, expectancies typically decrease (Sher et al. 1996); (5) when tested with statistical methods for demonstrating mediation, expectancies can be shown to mediate the influence of other known antecedents of drinking risk (Finn et al. 2000); and (6) expectancies can be manipulated in true experiments, with consequent changes in drinking levels (Roehrich and Goldman 1995).

Despite the extensive body of research that has related the expectancy concept to alcohol (and other drug) use and abuse, precise processes have only recently begun to emerge. One approach has been to treat expectancies as the primary content of memory and then to use memory models and methods as a basis for examining expectancy operation. This effort has made use of empirical models of memory networks and associated mathematical techniques (e.g., multidimensional scaling and clustering) to depict these networks (Rather et al. 1992). Based on these models, experiments have been performed using well-established cognitive paradigms (e.g., implicit and explicit stimulus priming, false memory techniques, Stroop task, free association), resulting in findings consistent with the empirically derived memory models (e.g., Kramer and Goldman 2003; Stein et al. 2000). Particularly noteworthy were studies showing that cognitive priming influences actual alcohol consumption (Roehrich and Goldman 1995; Stein et al. 2000). Such approaches are only just beginning to be applied to the development of behavioral approaches to reduce the risk for excessive alcohol use and abuse.

## Influential Theories of the Development of Adolescent Problems with Alcohol and Other Substances

The theories outlined below are among the historically most influential in research on the origins and progression of problems in adolescents with alcohol and other drugs. These theories have tended to be directed toward antisocial and deviant involvement with alcohol and other drugs; they fail to address the underage drinking behavior of youth thought to be successful and mainstream. It is the goal of NIAAA’s underage drinking initiative to stimulate the synthesis and testing of new and comprehensive models that reflect the complex multi-causality of all underage drinking behavior within a developmental framework.

### The “Gateway,” or Stage Theory

This theory comes from epidemiological research that has examined patterns of alcohol and other drug use progression among adolescents. The original findings suggested that adolescents initially experiment either with alcohol and/or cigarettes (as legal and culturally accepted drugs) and then progress to marijuana. Once experience with marijuana as an illicit drug is acquired, adolescents may then try other illicit drugs such as heroin and cocaine. The experimentation with alcohol and tobacco, as legal drugs, is viewed as a necessary intermediate or “gateway” to illicit drug use (Kandel and Faust 1975).

Opponents of this theory suggest that there may be a common risk factor (or factors) for illicit drug use that could account for the relationship between marijuana and other drug use independent of initial exposures to alcohol and tobacco. Examples of a theorized “third factor” include a genetic predisposition to problematic involvement with alcohol and other drugs, a predisposition toward adolescent high-risk behavior in general, or shared opportunities to obtain both marijuana and other drugs (Morral et al. 2002).

A less controversial offshoot of this theory deals with the age of first experimentation with a broad class of abused drugs (whether it includes alcohol, tobacco, marijuana, or hard drugs), and the timing of stages of regular use and problematic involvement. The literature converges around the association of early alcohol and other drug use, including underage drinking, and subsequent problematic involvement (Choi et al. 2001; Choi et al. 1997; Kandel and Logan 1984; Schuckit and Russell 1983; Yamaguchi and Kandel 1984; Hawkins et al. 1997; Grant and Dawson 1998; Warner and White 2003). For this reason, substantial attention has been focused on prevention interventions that delay the initiation of alcohol and other drug use.

### Problem Behavior Theory

Problem behavior theory is an influential conceptual framework for understanding not only problematic alcohol and other drug use but also a wide variety of high-risk adolescent behaviors (Jessor and Jessor 1977). The theory proposes a syndrome of comorbid adolescent problem behaviors that may manifest themselves within the same person (Jessor 1991). For example, adolescents with alcohol problems may engage in a spectrum of problem behaviors, such as illicit drug use, delinquent behaviors (e.g., truancy, petty theft, vandalism, lying, running away), risky and precocious sexual activity, and other high-risk behaviors (e.g., drag racing, drunk driving).

A single, possibly genetic, factor is hypothesized to underlie a general syndrome of deviance that may predate adolescence and persist into adult life*.* Developmental support for this conceptualization comes from the findings of the Dunedin Multidisciplinary Health and Development Study. In one group of adolescents in this study, significant conduct problems were observed in preadolescence, adolescence, and later adulthood (life-course-persistent antisocial behavior). Another adolescent group was characterized as having behavioral deviancy that began and ended during adolescence (adolescence-limited antisocial behavior). Adolescents in this group ultimately had a positive adult outcome (Moffit 1993).

### Comorbidity Theory

Epidemiological research has demonstrated significantly elevated rates of alcohol and other drug use problems among adolescents with defined psychiatric disorders. Most, but not all, clinical and community studies suggest that psychiatric disorders precede the development of adolescent alcohol and other drug use problems in these people (e.g., Ellickson and Hays 1991; Boyle et al. 1992; Van Kammen and Loeber 1994; Kellam and Anthony 1998). Some studies have found that psychiatric symptoms emerge during or after problematic involvement with alcohol and other drugs (e.g., Brook et al. 1998; Fergusson and Horwood 1996). Developmental research has suggested that the initial subsyndromal symptoms of most psychiatric disorders (except depression) precede the onset of adolescent alcohol and other drug use in people with both. Over time, these people receive full psychiatric diagnoses and subsequently progress to substance use and a substance use disorder diagnosis (Costello et al. 1999). The childhood psychiatric disorders known as the disruptive behavior disorders (i.e., attention deficit hyperactivity disorder, oppositional defiant disorder, and conduct disorder) have the strongest associations with problematic involvement with alcohol, tobacco, and other drugs during adolescence. It is noteworthy that among these, adolescent conduct disorder increases the risk for alcohol abuse and dependence (Deykin et al. 1987; Disney et al. 1999; Lewinsohn et al. 1993; Moss and Lynch 2001).

Depression also is associated with problematic involvement with a wide variety of alcohol and other drugs during adolescence (Kandel et al. 1997). Some anxiety disorders, such as separation anxiety, may be protective against alcohol and other drug use behavior during adolescence, whereas others, such as generalized anxiety disorder, may enhance alcohol drinking and augment risk (Kaplow et al. 2001). Adolescents with comorbid psychiatric disorders present a significant challenge for both prevention and treatment of adolescent alcohol problems.

### Maladaptive Coping Theory

High-risk behaviors also may be adaptive to the extent that they serve a social or maturational goal such as separating from parents, achieving adult social status, or gaining peer acceptance (Spear 2000). Engagement in high-risk behaviors may help an adolescent cope with failure, boredom, stress, social anxiety or isolation, unhappiness, rejection, and low self-esteem. One example of maladaptive coping is an adolescent’s reported use of alcohol and other drugs as a means of gaining social status and acceptance from peers and, at the same time, enhancing mood and assuaging feelings of low self-worth (DuRant 1995; DuRant et al. 1995). Thus, problematic involvement with alcohol and other drugs for some adolescents may be a maladaptive means of coping with the stresses and social pressures that are characteristic of the adolescent stage of development, particularly in the absence of adult support, guidance, and monitoring.

### Patterson’s Developmental Theory

This theory was originally proposed to explain the development of juvenile delinquency; however, consistent with the observation that problem behaviors frequently co-occur in adolescents, it also has been used to understand and address problematic involvement with alcohol and other drugs of abuse. Patterson and colleagues (Dishion et al. 1991; Patterson et al. 1989) are proponents of a developmental theory of conduct problems which assumes that adolescent problem behavior is a consequence of poor parental family management practices interacting with the child’s own aggressive and oppositional temperament. Here, temperament refers to the early and genetically determined behavioral characteristics that, over time and life experiences, evolve into personality. Deficits in parenting skill, such as harsh and inconsistent punishment, increased parent-child conflict, low parental involvement, and poor parental monitoring, result in school behavior and performance problems. The poorly performing and poorly behaving child may be socially rejected by average children; however, he or she forms close friendships with other problematic children. This process of forming close peer relationships is augmented by the negative interactions with caregivers in the home. As the child affiliates with more deviant children, he or she adopts deviant behavior as a norm and becomes less involved in home life. Other deviant children become powerful social role models; the child learns from them further deviant and socially unacceptable behavior, including experimentation with alcohol and other drugs. Early experimentation consistently has been found to be a risk factor for later problematic involvement with a wide variety of drugs.

These children, therefore, may be viewed as being on a developmental pathway of deviancy and alcohol and other drug abuse that begins in infancy and is compounded by unskilled parenting and the formation of social relationships with other problem children (Vuchinich et al. 1992). Prevention interventions that are based on this theoretical approach offer parenting skills training to teach parents more effective ways to discipline and monitor their children and to reduce the negative environment of the home. Tutoring and other forms of educational support may be provided to reduce academic failure. Social skills training also may be offered to the child to help him or her cope with rejection by normal peers and to provide a mechanism for graceful resistance to peer pressure to use alcohol and illicit drugs.

## Footnotes

1 Expectancies are stored information (memories) that anticipate future events.
